# Novel Ultrafiltration Polyethersulfone Membranes Blended with Carrageenan

**DOI:** 10.3390/polym17020176

**Published:** 2025-01-13

**Authors:** Saeed H. Al Marri, Yehia Manawi, Simjo Simson, Jenny Lawler, Viktor Kochkodan

**Affiliations:** Qatar Environment and Energy Research Institute, Hamad Bin Khalifa University, Qatar Foundation, Doha P.O. Box 34110, Qatar; saealmarri@hbku.edu.qa (S.H.A.M.); ssimson@hbku.edu.qa (S.S.);

**Keywords:** ultrafiltration membrane, polyethersulfone, carrageenan, water flux, water treatment, ultrafiltration

## Abstract

The development of ultrafiltration (UF) polymeric membranes with high flux and enhanced antifouling properties bridges a critical gap in the polymeric membrane fabrication research field. In the present work, the preparation of novel PES membranes incorporated with carrageenan (CAR), which is a natural polymer derived from edible red seaweed, is reported for the first time. The PES/CAR membranes were prepared by using the nonsolvent-induced phase separation (NIPS) method at 0.1–4.0 wt.% CAR loadings in the casting solutions. The use of dimethylsulfoxide (DMSO), which is a bio-based and low-toxic solvent, is reported. Scanning electron microscopy, atomic force microscopy, water contact angle, porosity, and zeta potential measurements were used to evaluate the surface morphology, structure, pore size, hydrophilicity, and surface charge of the prepared membranes. The filtration performance of PES/CAR membranes was tested with bovine serum albumin (BSA) solutions. It was shown that CAR incorporation in the casting solutions notably increased hydrophilicity, porosity, pore size, surface charge, and fouling resistance of the prepared membranes compared with plain PES membranes due to the hydrophilic nature and pore-forming properties of CAR. The PES/CAR membranes showed a significant reduction in irreversible and total fouling during filtration of BSA solutions by 38% and 32%, respectively, an enhancement in the flux recovery ratio by 20–40%, and an improvement in mechanical properties by 1.5-fold when compared with plain PES membranes. The findings of the present study indicate that CAR can be used as a promising additive for the development of PES UF membranes with enhanced properties and performance for water treatment applications.

## 1. Introduction

Ultrafiltration is widely employed in various fields including water treatment as well as separation of biological macromolecules including proteins, food processing, biotechnology, and others. An efficient UF membrane is a key element for successful UF applications. Polyethersulfone (PES), which possesses high chemical, mechanical, and thermal resistance properties, is one of the polymers most often used for the fabrication of UF membranes [1,2,3]. On the other hand, PES is rather hydrophobic and characterized by high water contact angle values ranging between 65° and 80° and low surface charge [3]. This results in PES membrane fouling especially when used for filtration of protein-containing solutions [4,5,6]. It has been well documented that membranes with hydrophobic surfaces and low surface charge are very prone to fouling with organic substances, colloids, and microorganisms [7,8]. UF membrane fouling is one of the main challenges that is widely reported to compromise the performance of the membranes as it reduces the permeate flux which is followed by a surge in the operational transmembrane pressure required to run the UF process [9]. This leads to higher energy consumption and a higher carbon footprint. In addition, more frequent chemical cleaning is required to maintain the performance of the fouled membranes which leads to a shorter membrane lifetime [10,11].

Asymmetric PES membranes are generally prepared using the phase-inversion technique and the membrane structure, properties, and performance notably depend on various parameters, such as the composition and temperature of a casting solution and coagulation bath [12]. One of the main approaches to adjusting the porous structure and hydrophilicity of PES UF membranes is through the incorporation of hydrophilic additives (organic or inorganic) in the PES membrane matrix during membrane casting [3,13,14]. PVP and PEG are the most used additives for casting PES membranes [15,16,17,18,19]. It has been reported that PVP usually induces thermodynamic instability and promotes instantaneous de-mixing in the dope solutions thus enhancing macrovoid formations in the cast membranes [15,16]. The use of PVP to modify PES membranes was reported to demonstrate higher water fluxes as well as higher molecular weight cutoff (MWCO) values for prepared membranes in addition to decreasing the membrane fouling during the filtration of BSA solutions [20].

The formation phenomena of macrovoids, which can be described as large elongated hollow structures below the upper surface of the membrane, have been extensively investigated by Smolders et al. [21] and Wang et al. [22]. It was observed that porous structure and the performance of PES membranes were notably affected by both molecular weight (MW) and concentration of PEG additives [12].

Recently, amphiphilic polymeric additives, which can locate themselves at the membrane–water interface due to the presence of hydrophilic segments, whereas the hydrophobic parts of macromolecules are firmly anchor in the polymer matrix, were suggested for casting PES membranes [23,24]. Loh et al. [25] found that hydrophilic segments of the amphiphilic additives segregated to the polymer–water interface, with the hydrophobic segment firmly anchored to the polymeric matrix. Manawi et al. [26] used amphiphilic acacia gum as a pore-forming agent for the preparation of PES membranes, which were prepared using the phase-inversion technique. It was shown that the addition of acacia gum notably increased the surface charge, hydrophilicity (by 25%), porosity (by 83%), and permeate flux (by 130%) compared with plain PES membranes. Najjar et al. [27,28] showed that the PES membranes incorporated with acacia gum, graphene oxide, and carbon nanotubes (CNTs) showed significant enhancement in their hydrophilicity, surface charge, porosity, water flux, and improved biofouling resistance compared with plain PES membranes.

In addition to organic additives, different inorganic materials, including TiO_2_, SiO_2_, Al_2_O_3_, Fe_3_O_4_, carbon nanotubes (CNTs), graphene oxide, alumina, and metal–organic frameworks have been incorporated in the PES membranes [13]. For example, at the same 2 wt.% loading of TiO_2_, SiO_2_, Al_2_O_3_, and Fe_3_O_4_ particles in dope solutions, the water contact angle (WCA) of the cast PES membranes decreased from 71.9° to 59.6°, from 78.6° to 58.1°, and from 62.22° to 49.27° [26], respectively.

CNTs are among the additives widely discussed for the synthesis of nanocomposite membranes, including PES membranes, for water treatment [29]. However, some studies showed a toxic effect of CNTs on human health [5] if they leach out of the membrane and reach the treated water.

**Figure 1:**
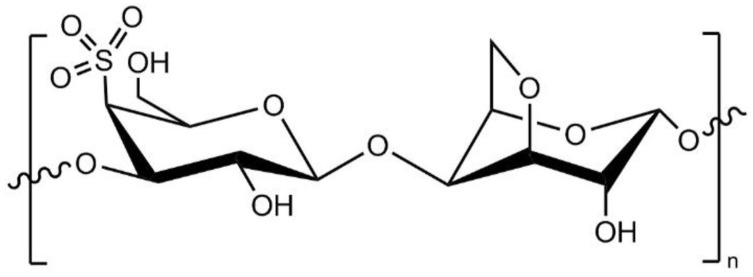
Chemical structure of CAR; adapted from [33].

In the present work, carrageenan (CAR), which is a natural polysaccharide derived from edible red seaweed [30], is used as an additive in preparations of PES membranes for the first time. As seen from its chemical formula in Figure 1, CAR is a highly hydrophilic compound bearing negatively charged sulfonic groups. The MW of CAR averages between 400 to 600 kDa [31]. It is used in many food-related applications, especially in sauces and culinary items, as a thickening, stabilizing, and gelling agent. It is also used in industrial operations, cosmetics, pharmaceutical formulations, and experimental medicine [30]. CAR has been used as a hydrophilic polymer in the development of polyvinylidene fluoride (PVDF) composite membranes. Adding CAR resulted in the formation of an asymmetric structure in the membrane as hydrophilicity increased in the PVDF/CAR composite [32]. The PVDF/CAR composite membrane had a smooth negatively charged surface and enhanced hydrophilicity, which caused strong resistance to dye adhesion and resulted in higher dye rejection and water permeability.

The main objective of this work is to study the effect of using CAR on the hydrophilicity, porous structure, surface charge, water flux, compaction, and fouling resistance of the PES/CAR membranes. To the best of our knowledge, the use of CAR to enhance the properties of PES has never been reported in literature.

## 2. Materials and Methods

### 2.1. Materials

PES flakes (molecular weight of 72,000 Da) were obtained from BASF (Ludwigshafen, Germany). Dimethyl sulfoxide (DMSO, 99%), CAR, and bovine serum albumin (BSA with molecular weight of 66.5 kDa) were purchased from Sigma-Aldrich (St. Louis, MO, USA). Millipore deionized water (DW) was used in the coagulation bath as a nonsolvent.

### 2.2. Methods

#### 2.2.1. Preparation of PES/CAR Membranes

**Figure 2:**
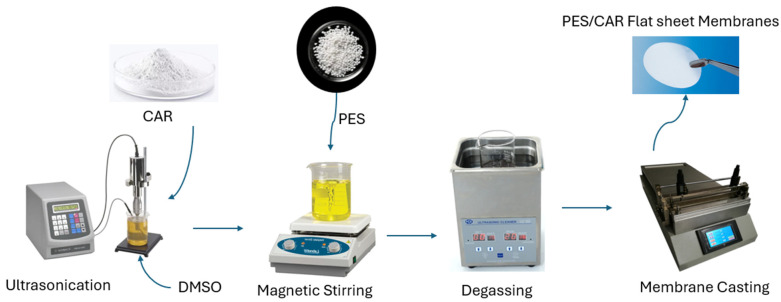
Schematic diagram of PES/CAR membrane preparation.

The preparation technique followed to develop PES/CAR membranes is depicted in the schematic diagram in Figure 2. The nonsolvent-induced phase separation method was used for preparation of PES membranes by using a flat sheet membrane casting system (Philos Co., Ltd., Gwangmyeong-si, Republic of Korea). PES/CAR membranes were cast at different loadings of CAR (0.1, 0.3, 0.5, 0.75, 1.0, 2.0, and 4.0 wt.%) in the 16 wt.% PES dope solution in DMSO (Table 1).

For the preparation of the casting solutions, the required amount of CAR was added to DMSO and sonicated using a Fisher Scientific ultrasonic process (Hampton, NH, USA) for 40–50 min at 60% intensity and pulses every 5 s. After that, PES was dissolved in the DMSO/CAR solution by mixing for 4–5 h at 60 °C using a magnetic stirrer. The dope solution was then degassed for about 6 h. After that, PES/CAR membranes were cast by using the nonsolvent-induced phase separation method at a casting speed of 20 cm/s at room temperature. The glass plate with the cast membrane film was then immersed in distilled water and kept until the membrane was detached from the glass plate. The membranes were then washed in deionized water for 24 h at room temperature.

#### 2.2.2. Membrane Characterization

The surface and cross-section morphologies of the membranes were analyzed using field emission scanning electron microscopy (FE-SEM) (FEI Quanta 650FEG SEM, FEI, Hillsboro, OR, USA) for imaging and Bruker Quantax400 EDS for microanalysis. The characterization was carried out under vacuum conditions at 5 kV. For the cross-section, membrane samples were fractured using liquid nitrogen and all the membrane samples were coated with 5 nm of gold before scanning for conductivity.

The gravimetric method was used to determine the total porosity of the produced membranes [15]. The porosity of each membrane was evaluated by taking three samples and calculating the average. The circular membrane samples were dried in an oven at 50 °C for 24 h before measuring their mass in dry weight. Then, the membrane samples were submerged in DW at a temperature of 25 °C for 24 h. Thereafter, the membrane samples were removed from DW, and DW was gently wiped off using filter paper before measuring the wet mass of the membrane samples. The total porosity of the membrane (*ε*) was evaluated by using Equation (1) [34].

(1) $$\varepsilon =\frac{w_{w}-w_{d}}{A\times l\times \rho }\times 100\%\;$$

where *w_w_* and *w_d_* are the wet and dry masses of the membranes, respectively; *ρ* is the density of the distilled water at 25 °C (998 kg/m^3^); *A* is the membrane surface area (m^2^); and *l* is the thickness of the membranes.

The Guerout–Elford–Ferry equation was used to evaluate the average pore radius (rm) of the membrane [17].

(2) $$r_{m}=\sqrt{\frac{\left(2.9-1.75\varepsilon \right)8\eta lQ}{\varepsilon A\Delta P}}$$

where *η* is the viscosity of water at 25 °C which is equal to 8.9 × 10^−4^ Pa s, *Q* is the permeate volume in m^3^/s, and Δ*P* is the operating pressure which is equal to 1 bar.

The hydrophilicity/hydrophobicity of the fabricated membranes was evaluated by measuring the contact angle between the membrane surfaces and water droplets, and the contact angle measurements were performed by using a Kruss DSA25 Drop Shape Analyzer (KRÜSS GmbH, Hamburg, Germany). The surface roughness of the membrane was determined using a Bruker Icon Dimension Atomic Force Microscope (AFM) (Billerica, MA, USA). The scan was performed over a 1 µm × 1 µm area for comparison purposes.

The measurement of zeta potential of CAR powder was carried out using Zetasizer Ultra (Malvern Panalytical, Malvern, Worcestershire, UK) at different pH values by using an MPT-3 multi-purpose titrator.

The determination of zeta potential of PES and PES/CAR membranes was performed using a SurPASS 3 electrokinetic analyzer (Anton Paar, Graz, Austria). In this work, the zeta potential of the membrane surfaces was measured at different pH values by adjusting the pH of the electrolyte solution using 0.1 M HCl and 0.1 M NaOH solutions.

The viscosity of the casting solutions was measured using a TA Instruments Discovery Hybrid Rheometer (TA Instruments, New Castle, DE, USA), utilizing a 40.0 mm parallel plate. A flow sweep experiment was conducted at 25 °C and 10 s soak time. Logarithmic sweep was performed at 100 s^−1^.

The analysis of the mechanical properties of the pure and modified membranes was conducted using a dynamic mechanical analyzer (DMA Q800, TA Instruments, New Castle, DE, USA), utilizing a film tension clamp. A displacement ramp experiment was conducted at a rate of 100.00 µm/min.

#### 2.2.3. Membrane Filtration Tests

Filtration tests were performed by using a dead-end stirred cell (HP4750X, Sterlitech, Kent, WA, USA) of 300 mL volume and the active membrane area of 14.6 cm^2^. Nitrogen gas was used for the pressurization of the cell.

The permeate flux (*J*) was evaluated using Equation (3):

(3) $$J=\frac{Q}{A\times T}$$

where *Q* is the permeate volume (L) collected through the membrane cross-sectional area *A* (m^2^) during filtration time *T* (h).

The rejection experiments were conducted using 300 mg/L BSA solution at pH 6.8 and an operating pressure of 1 bar. The permeate flux was collected for 45 min. The BSA rejection (*R*) was calculated using Equation (4):

(4) $$R=\left(1-\frac{C_{p}}{C_{f}}\right)\times 100\%$$

where *C_p_* and *C_f_* are the BSA concentrations in permeate and feed solution, respectively.

BSA concentration in water samples was evaluated by measuring the optical density of the solutions at 220 nm wavelength by using a Shimadzu spectrophotometer (Shimadzu Corporation, Kyoto, Japan).

#### 2.2.4. Antifouling Tests with BSA Solutions

The membrane resistance to organic fouling was evaluated with filtration of 300 mg/L BSA solutions. Before filtration of BSA solutions, DI water was filtered through the membrane for 30 min. After that, the membrane cell was loaded with 300 mg/L of BSA solution at pH 6.8 and permeate flux was collected for 45 min. Next, the membrane was rinsed with DI water for 5 min, then the cell was filled with DI water, and water flux was measured again for 30 min to estimate the water flux recovery. The filtration experiments were performed at an operating pressure of 1 bar.

The flux recovery rate (*FRR*) of the membranes after BSA filtration was determined using Equation (5):

(5) $$FRR=\left(\frac{J_{2}}{J_{1}}\right)\times 100\%$$

where *J*_1_ and *J*_2_ denote the initial and final DI water flux after filtration of BSA solutions for 45 min. 

The values of *J*_1_, *J*_2_, and BSA flux (*J_BSA_*) at the end of the BSA filtration experiments were used to calculate the total fouling rate (*R_t_*), reversible fouling rate (*R_r_*), and irreversible fouling rate (*R_ir_*) using Equations (6)–(8) [35]:

(6) $$R_{r}=\left(\frac{J_{2}-J_{\mathrm{BSA}}}{J_{1}}\right)\times 100\%$$

(7) $$R_{ir}=\left(\frac{J_{1}-J_{2}}{J_{1}}\right)\times 100\%$$

(8) $$R_{t}=R_{r}+\;R_{ir}$$

The values of *J*_1_ and *J*_2_ were measured both ahead and after the BSA filtration experiment, which lasted for 30 min.

The aforementioned performance testing was conducted three times, with each iteration using a new membrane, in order to verify that there were no notable discrepancies between the findings.

#### 2.2.5. Membrane Compaction Tests

The compaction factor (*CF*) of the membranes was determined at 1 bar using Equation (9):

(9) $$CF=\frac{J_{o}}{J_{f}}$$

where *J_o_* and *J_f_* stand for the initial DI water flux and DI water flux after 45 min, respectively.

## 3. Results and Discussion

### 3.1. Characterization of CAR and PES/CAR Membranes

**Figure 3:**
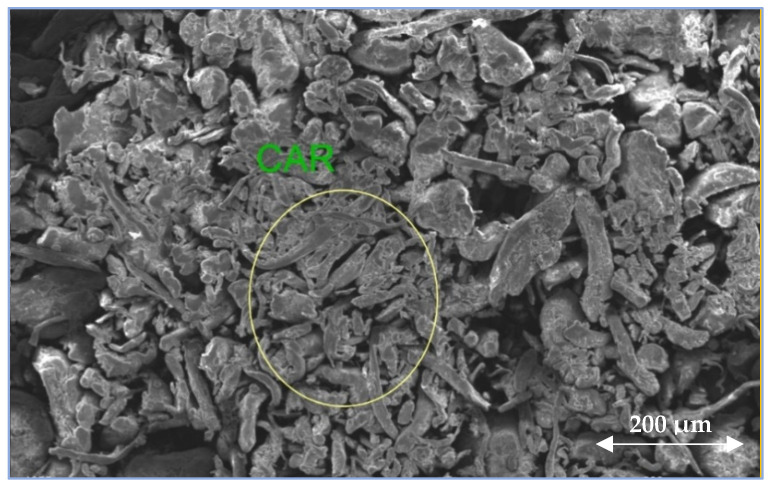
SEM image of CAR powder.

**Figure 4:**
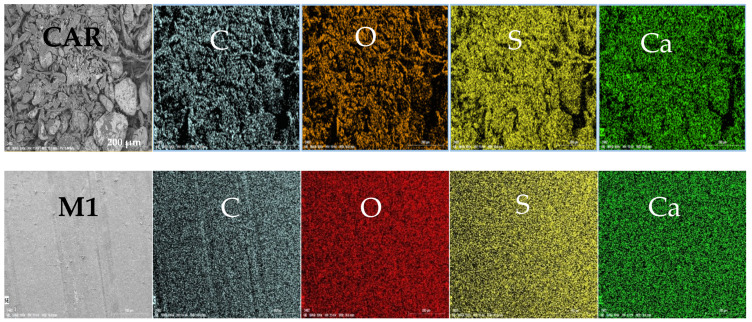

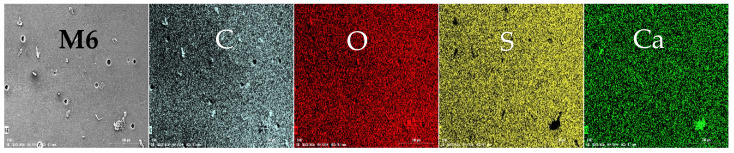
SEM mapping depicting the main elements present in CAR as well as M1 (0 wt.% CAR), M4 (0.5 wt.% CAR), and M6 (1 wt.% CAR) PES membranes.

Figure 3 shows the SEM image of CAR used in the present work. Moreover, the SEM mapping of the main elements present in CAR as well as M1, M4, and M6 PES membranes are depicted in Figure 4. The SEM mapping analysis showed that the main components present in PES/CAR membranes are the following: carbon, oxygen, sulfur, and calcium.

Moreover, the energy-dispersive spectrometer (EDS) was used to perform the elemental composition of CAR and PES/CAR membranes. The mass percentages of the main elements such as carbon, oxygen, sulfur, and calcium in CAR are 15.87%, 20.26%, 4.23%, and 1.89%, respectively. The EDS analysis conducted by Iqbal et al. [36] was observed to be in good agreement with the work reported in the present work. Moreover, the investigation of the chemical composition of CAR conducted by Tuvikene et al. [37] showed that CAR naturally contains calcium (2.7%), potassium (6.1%), and sodium (0.59%). The high levels of potassium and calcium were reported to be critically essential and highly desirable for the gelation of kappa CAR. The level of sulfur in CAR analyzed using the EDS technique in the present work was found to account for 6% mass, which is close to the level of 7% reported by Tuvikene et al. [37]. The mass percentage of sulfur in the pure PES membrane was 14% while that in the M5 membrane was 14.13%. It is worth pointing out that the higher sulfur content in the PES/CAR membranes compared to the pure membrane is due to the presence of sulfate groups in CAR macromolecules. The presence of sulfate groups in the modified membranes can lead to higher negative surface charge and hence increases the repulsion with negatively charged foulants and minimized membrane fouling.

**Figure 5:**
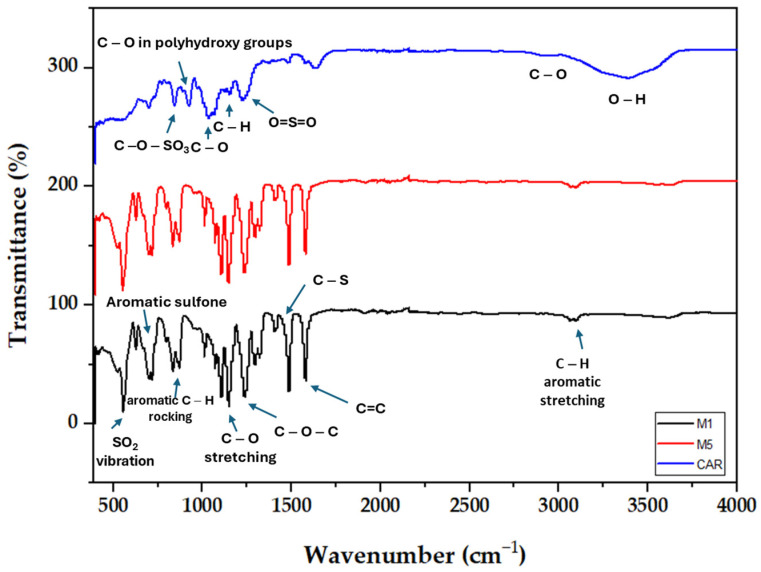
FTIR spectra of carrageenan, M1 (pure PES), and M5 (0.75 wt.% CAR) membrane samples.

The FTIR spectra of CAR are depicted in Figure 5. As seen, the presence of the band corresponding to the O–H stretching vibration was found to occur at 3400 cm^−1^. Moreover, the existence of the bridge C–O stretching vibration was also observed to occur at around 2920 cm^−1^, respectively. The analysis of the FTIR spectra of CAR showed the presence of the stretching vibration of C–H at 1155 cm^−1^ and C–O stretch at 1033 cm^−1^. Moreover, the symmetric vibration of O=S=O was also found to occur at 1223 cm^−1^. Furthermore, the presence of stretching vibrations of C–O–SO_3_ in d-galactose-4-sulfate as well as C–O in polyhydroxy groups at 844 and 925 cm^−1^ was also found to exist in CAR. The FTIR spectra of CAR in the present work were found to be in good agreement with the FTIR studies reported by Ulu et al. [38], Fan et al. [39], Silva et al. [40], Liew et al. [41], and Abdullah et al. [42].

Moreover, bare PES and PES/CAR membranes were analyzed using the FTIR technique. Figure 5 shows the FTIR spectra of M1 and M5 samples. As seen, both membranes showed almost identical FTIR spectra that might be explained by the low CAR loading in the modified membrane. The existence of SO_2_ vibration as well as aromatic C–H rocking vibration was observed at 560 and 835 cm^−1^, respectively [42,43,44,45]. Aromatic sulfone functional groups’ peaks are reported to occur at frequencies ranging between 700 and 831 cm^−1^ [43,44,45,46]. The C–O stretching vibration was also observed in both membranes at a wavelength of 1150 cm^−1^ [47]. The stretching vibration of C–O–C was also found to occur at a wavelength of 1240 cm^−1^ [48]. The peaks found at 1484 and 1581 cm^−1^ corresponded to C–S and C=C ring vibration functional groups [49]. Furthermore, the peak located at 3086 cm^−1^ in both membranes was cited in the literature to be C–H aromatic stretching vibrations [44].

### 3.2. Zeta Potential of CAR

**Figure 6:**
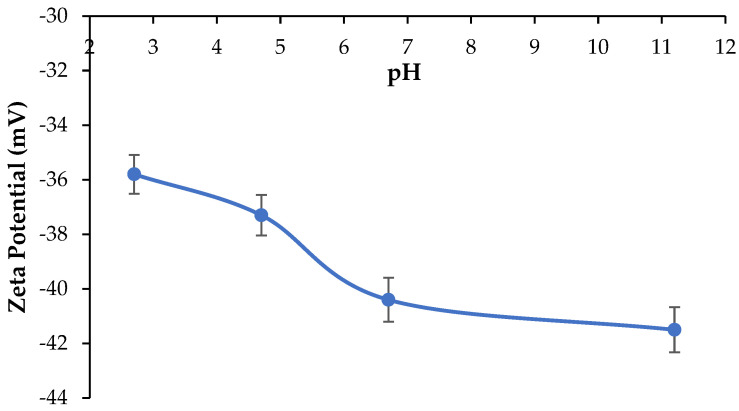
Zeta potential of CAR macromolecules at different pH values.

Figure 6 shows the zeta potential of CAR at acidic, neutral, and basic conditions. The pH was adjusted by adding 0.05 M aqueous solutions of HCl and NaOH. As observed, CAR showed negative zeta potential values throughout the pH range investigated in the present work from −35.8 ± 1.0 mV at pH 2.5 to −41.5 ± 1.4 mV at pH 11. Furthermore, the zeta potential at neutral pH (~7) was −40.4 ± 1.3 mV. The zeta potential of CAR reported by Guo et al. [50] ranged between −52 mV at pH 2 and −75 mV at pH 8 which was more negative than the zeta potential values reported in the present study. The negative zeta potential of CAR macromolecules is attributed to the presence of sulfonic groups which are negatively charged in water at the studied pH range [49,50,51].

### 3.3. Membrane Morphology and Porosity

**Figure 7:**
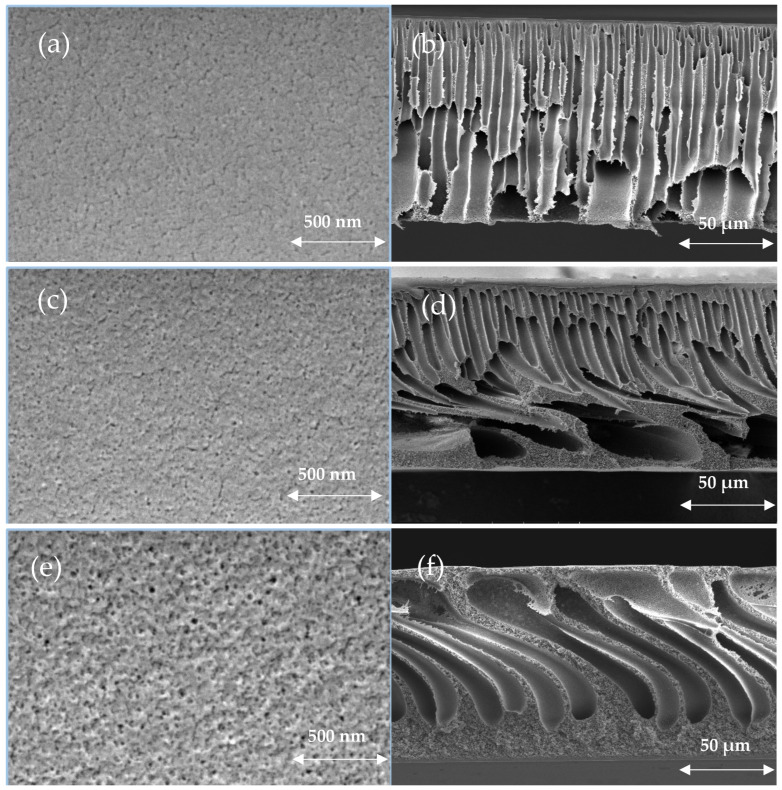
SEM images of the surface (**a**,**c**,**e**) and cross-section (**b**,**d**,**f**) of plain PES (**a**,**b**), M6 (1 wt.% CAR) (**c**,**d**), and M8 (4 wt.% CAR) (**e**,**f**) membranes.

The morphology of the prepared membranes was investigated by analyzing the top membrane surface and the membrane cross-section by the SEM technique. Figure 7a shows the top view of the plain PES membranes (without CAR) while Figure 7b shows PES membranes incorporated with 4 wt.% CAR under the same magnification. Moreover, the cross-sections of the pure as well as the modified membrane (4 wt.% CAR) are depicted in Figure 7c,d. Both membranes were observed to show a thin top layer in addition to a sponge-like structure in their cross-sections. However, as seen in Figure 7, the effect of adding CAR on the structure of PES membranes can be confirmed by observing the difference in the structures of both membranes. As seen, the modified membranes demonstrated the formation of larger finger-like macrovoid structures within the modified membranes compared to the pure PES membrane.

Obviously, the addition of CAR, which acts as a nonsolvent medium due to its high water solubility, facilitates the immediate de-mixing process between solvent and nonsolvent during membrane casting that results in the formation of large finger-like macrovoids within the membrane matrix. Overall, the addition of CAR to PES membranes was observed to follow the same trend cited in the literature when natural-based additive was added to casting solutions of polymeric membranes [26].

**Figure 8:**
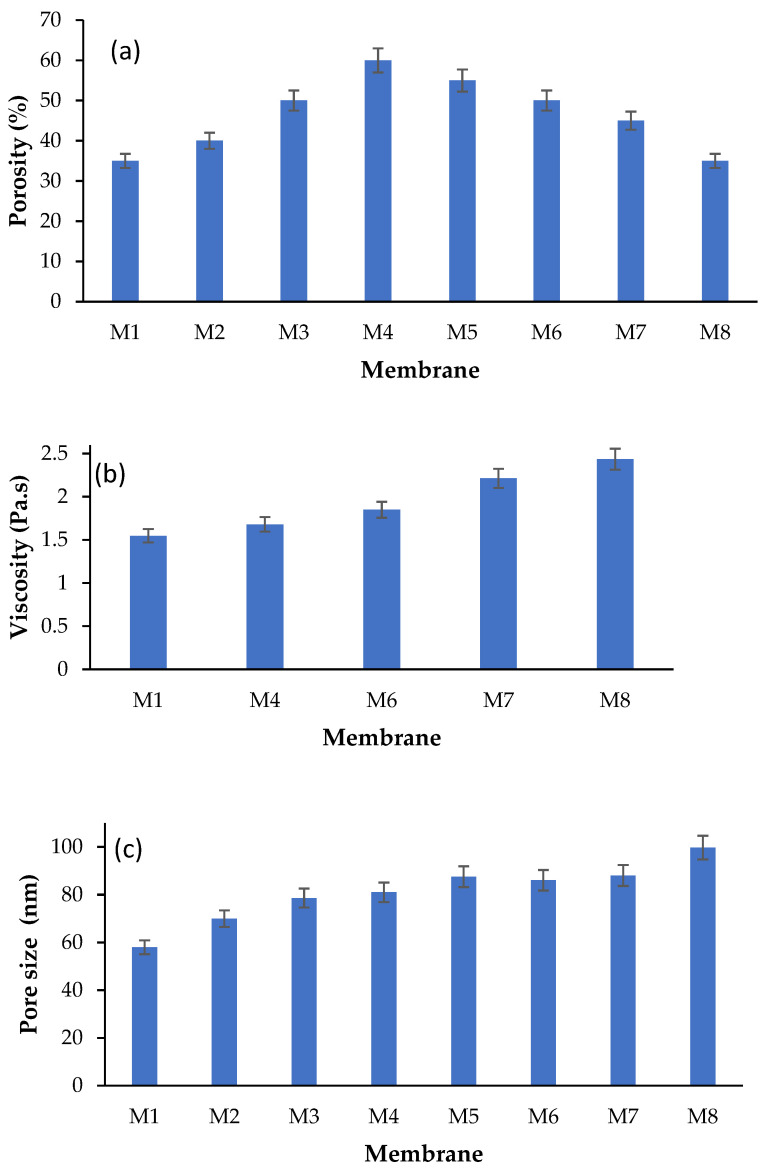
Average total porosity (**a**) and pore size (**b**) of PES membranes as well as viscosity (**c**) of casting solutions at various loadings of CAR in casting solutions; temperature: 25 °C, shear rate: 100 1/s. (M1: pure PES, M2: 0.1 wt.%, M3: 0.3 wt.%, M4: 0.5 wt.%, M5: 0.75 wt.%, M6: 1 wt.%, M7: 2 wt.%, and M8: 4 wt.% CAR).

The total porosity of the membranes prepared in the present work was calculated using Equation (1) and the values are shown in Figure 8a. The SEM images depicted in Figure 7 agree well with porosity measurements shown in Figure 8a. As seen, the increase in the loading of CAR was found to enhance the total porosity from 35 ± 1.5 (%) (pure PES membrane) up to 60 ± 4.5 (%) at a CAR loading of 0.5 wt.% in the dope solution. This increase can be attributed to the enhanced interchange between solvent and nonsolvent during phase inversion which formed membranes with a more porous structure [26,52,53]. The increase in the loading of CAR beyond 0.5 wt.% was observed to result in lowering the overall porosity of the membranes, obviously due to the increase in the viscosity of the casting solutions.

The viscosity of the casting solutions at various loadings of CAR onto PES was investigated at a temperature of 25 °C and shear rate of 100 1/s (Figure 8b). As seen, the addition of CAR was observed to increase the viscosity of the casting solutions of the pure membrane from 1.5 ± 0.2 to 2.4 ± 0.15 Pa·s after the addition of 4 wt.% of CAR. The viscosity increase was reported to decelerate the process of polymer coagulation and lead to the formation of denser and less porous membranes [26,52,53].

The changes in calculated membrane porosity values were observed to correlate with the changes in morphological structure of the membranes investigated using the SEM technique which showed higher membrane porosity after the incorporation of CAR (Figure 7). 

The findings of this study correlate well with previously published data. For example, Alam et al. [32] showed increasing membrane porosity for PVDF UF membranes incorporated with 2 wt.% CAR compared to the pure PVDF membrane from 22 to 60%.

Moreover, the authors reported the increase in the loading of CAR in the casting solutions increased the viscosity of the solutions and hence changed the structure and morphology of developed PVDF/CAR membranes. The authors correlated the change in viscosity to enhanced intermolecular aggregation as well as rigorous chain entanglement [32].

In the present work, the addition of CAR was observed to enhance the porosity of the modified membranes up to the highest level corresponding to M4 which contains 0.5 wt.% CAR in the casting solution. The increase in CAR loading beyond 0.5 wt.% was found to reduce the average porosity of the membranes due to the rise in the casting solutions’ viscosity that suppressed the rapid de-mixing process in a coagulation bath. This was found to agree with work reported in the literature when acacia gum (AG) was added to dope PES solutions and it was found that the membrane porosity decreased beyond the AG loading of 1 wt.%, which was confirmed by SEM images depicting the formation of a thick top layer with poor pore connection.

Moreover, Figure 8c demonstrates that the pure PES membrane had an average pore size of 58.1 ± 3.2 nm, whereas the pore sizes for PSE/CAR membranes range from 70.2 ± 3.9 to 99.7 ± 4.6 nm.

### 3.4. Membrane Hydrophilicity

**Figure 9:**
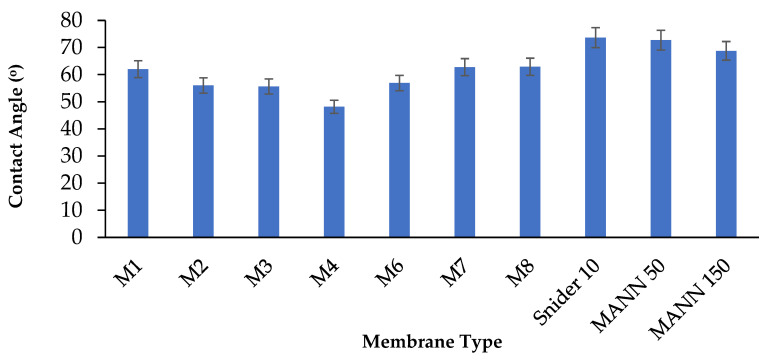
Water contact angle of PES membranes incorporated with various loadings of CAR in casting solutions and commercial PES membranes with molecular weight cut-off of 10 kDa (Snider) and 50 and 150 kDa (Mann+Hummel). (M1: pure PES, M2: 0.1 wt.% CAR, M3: 0.3 wt.% CAR, M4: 0.5 wt.% CAR, M6: 1 wt.% CAR, M7: 2 wt.% CAR, and M8: 4 wt.% CAR).

The hydrophilicity of the membranes was investigated by evaluating the contact angle between the water droplet and membrane surface. Figure 9 shows the water contact angle of PES membranes at various loadings of CAR. As seen, the addition of hydrophilic CAR was observed to increase the hydrophilicity by reducing the water contact angle. For instance, the water contact angle of the pure PES membrane was 62 ± 4.3° and the value was found to decrease to 48 ± 3.5° at a CAR loading of 0.5 wt.%. The enhancement in the membrane hydrophilicity can be attributed to the amphiphilic nature of CAR macromolecules which anchor the hydrophobic sites to the PES membrane surface while the hydrophilic segments end at aqueous solutions. It is worth pointing out that the hydrophilicity of the PES membrane at a CAR loading of 0.5 wt.% was higher than the commercial membranes Snider (PES 10 kDa) and Mann+Hummel (PES 50 kDa and PES 150 kDa), as confirmed by the water contact angle values shown in Figure 8. Commercial PES membranes are hydrophobic in nature and are prone to solute adsorption from various feed streams.

The enhanced membrane hydrophilicity was reported in the literature to play a crucial role in minimizing membrane fouling and maintaining the performance of the membranes during filtration due to the increased interaction between water and hydrophilic membrane surfaces as well as the reduction in the interaction between hydrophobic foulants and hydrophilic membrane surfaces [8,11,29,54,55,56,57,58].

The increase in the loading of CAR beyond the optimum loading of 0.5 wt.% was observed to reduce the hydrophilicity of the membranes, obviously due to the possible agglomeration of CAR in the dope solutions, which was reported to reduce the hydrophilization of membrane surfaces above an optimum loading [26].

### 3.5. Membrane Surface Roughness

**Figure 10:**
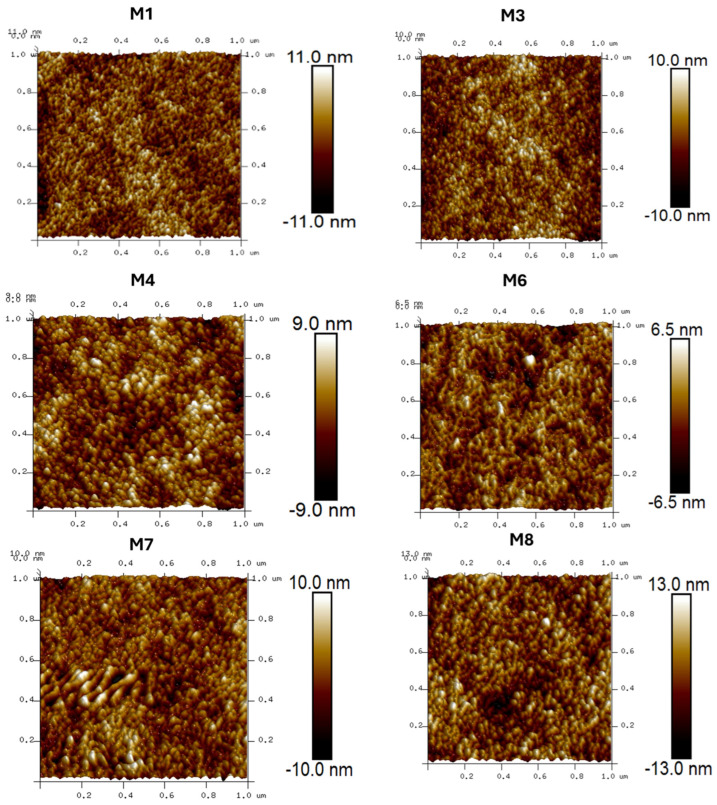
Images depicting 1 µm scan area of PES membranes doped with various loadings of CAR onto casting solutions. (M1: pure PES, M3: 0.3 wt.% CAR, M4: 0.5 wt.% CAR, M6: 1 wt.% CAR, M7: 2 wt.% CAR, and M8: 4 wt.% CAR).

The effect of adding CAR to the casting solutions on the surface roughness of the prepared PES membranes was investigated by using the AFM technique. Table 2 lists the average surface roughness while Figure 10 depicts the 1 µm scan area corresponding to the investigated membranes. It was found that 1 wt.% CAR loading in the casting solution was observed to reduce the surface roughness from 2.46 ± 3.11 nm for the pure PES membrane to 1.44 nm for the M4 sample. As seen, the pure PES membrane is characterized with ridge–valley structures while the PES/CAR membranes showed smoother membrane surfaces. The reduction in the surface roughness of the modified PES membranes with the increase in CAR loading up to 1 wt.% is obviously due to the interaction between PES and CAR macromolecules that minimize formation of ridge–valley structures as a result of development of closely packed and more intense entanglements [32]. Conversely, the increase in the loading of CAR beyond the optimum loading of 1 wt.% was observed to increase the average surface roughness from 1.44 ± 2.68 nm to 2.98 ± 3.74 nm due to the development of scattered nodules as well as microvilli-type formations [32]. The reduction in the surface roughness of the membranes is important for the reduction in the attachment of colloids to the membrane surface and hence reduces the membrane fouling [8].

### 3.6. Zeta Potential of Membranes

**Figure 11:**
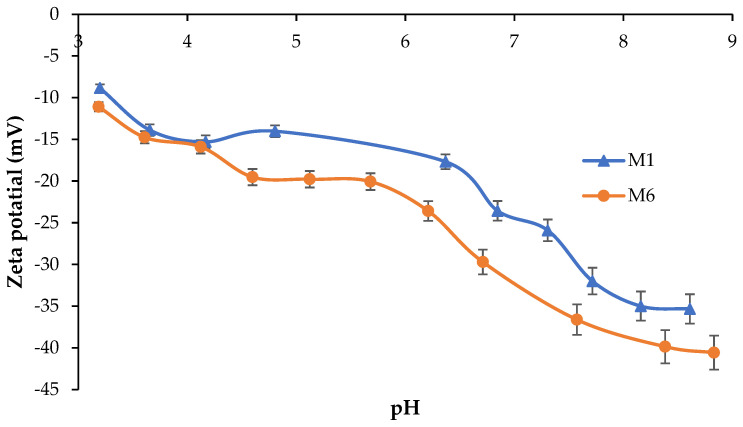
Zeta potential of pure PES (M1) and the modified PES membrane with 1 wt.% CAR in the dope solution (M6).

The surface charge of the prepared membranes was measured using the Anton Paar Surpass 3 electrokinetic analyzer at acidic, neutral, and basic pH (3–9). Zeta potential is generally employed in order to quantify the surface charge of a membrane’s surface at different pH values. Figure 11 illustrates the zeta potential values of the pure PES membrane (M1) as well as the M6 sample prepared with 1 wt.% of CAR in the dope solution. As seen, the zeta potential of the pure PES membranes decreases along with the increase in the solution pH due to the adsorption of OH^−^ on the surface of the membranes following the addition of NaOH for pH adjustment [54,59].

Moreover, the zeta potential of the modified membrane was observed to be more negative when compared with the plain PES membrane. This is attributed to the presence of sulfonic groups in CAR macromolecules embedded in the membrane matrix, which are dissociated at the studied pH range.

Similarly, it was reported that the zeta potential of PES membranes modified with acacia gum bear more negativity compared with the pure PES membrane due to dissociation of carboxylic groups of acacia gum embedded in the membrane matrix [26]. The modified membranes characterized with higher negatively charged surfaces and enhanced hydrophilicity were reported in the literature to enhance the antifouling resistance during filtration of solutions containing various types of foulants such as bacteria, proteins, etc. [26].

### 3.7. Mechanical Properties

**Figure 12:**
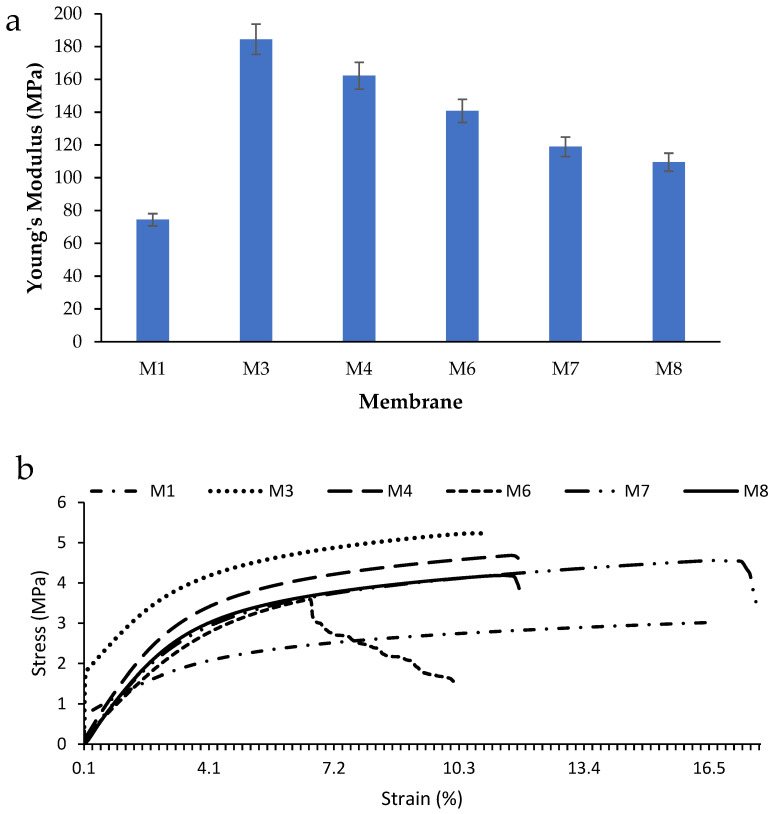
(**a**) Young’s modulus and stress vs. strain curve (**b**) for various PES/CAR membranes. (M1: pure PES, M3: 0.3 wt.% CAR, M4: 0.5 wt.% CAR, M6: 1 wt.% CAR, M7: 2 wt.% CAR, and M8: 4 wt.% CAR).

The effect of incorporating CAR onto the mechanical properties of PES membranes was investigated by dynamic mechanical analysis (DMA). Figure 12 shows the correlation between Young’s modulus (*E*), which estimates the tensile strength of the membranes, and the loading of CAR in the casting solutions. The Young’s modulus can be determined by dividing the stress over the produced strain. As observed, the incorporation of CAR was found to enhance the Young’s modulus of the pure membrane from 74.4 ± 3.2 MPa until it reaches the highest Young’s modulus of 184.4 ± 10.4 MPa corresponding to the M3 sample (0.3 wt.% loading of CAR). The enhancement in the mechanical properties of PES/CAR membranes was reported in the literature to take place due to the anchoring of amphiphilic macromolecules to the hydrophobic end of the PES polymer, which results in increasing the tensile strength of the modified membranes [34]. The increase in the loading of CAR beyond 0.1 wt.% was observed to reduce Young’s modulus due to the agglomeration of excess CAR and increase in the fragility of the membranes. The enhancement in the mechanical properties following the addition of CAR in the present work was found to outperform the work reported in the literature [26,56,60]. For instance, the addition of hydrophilic surfactant (Tween-20) as well as lipophilic surfactant (Span80) to the casting solutions of polysulfone membranes was reported by Tsai et al. [59]. They reported a decrease in the Young’s modulus of the membranes from 32.7 to 13.3 MPa following the addition of 15 wt.% of Tween-20 in the casting solutions, whereas the addition of 15 wt.% of Span80 to the casting solutions increased the Young’s modulus of the membranes from 32.7 to 49.2 MPa. The authors attributed the reduction in Young’s modulus with the addition of Tween-20 to the plasticizing impact of the hydrophilic surfactant. Conversely, the addition of Span80 resulted in the modification of the structure of the prepared membranes by restraining the development of macrovoids as well as the promotion of the formation of thick and dense membranes.

### 3.8. Filtration Experiments with PES/CAR Membranes

#### 3.8.1. Water Flux

**Figure 13:**
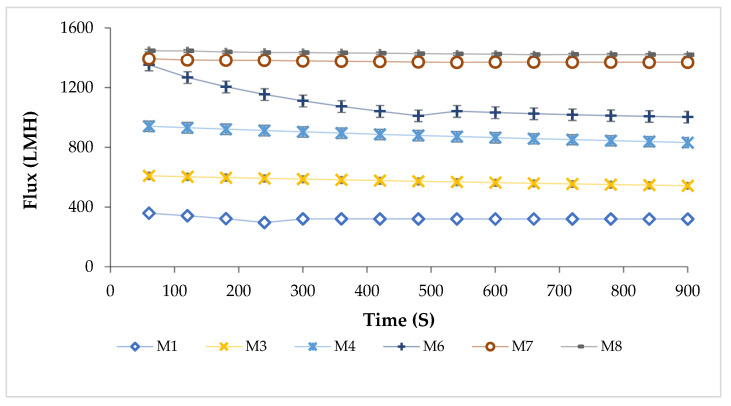
Pure water flux of PES membranes incorporated with various loadings of CAR in casting solutions; TMP: 1 bar. (M1: pure PES, M3: 0.3 wt.% CAR, M4: 0.5 wt.% CAR, M6: 1 wt.% CAR, M7: 2 wt.% CAR, and M8: 4 wt.% CAR).

The water filtration experiments were conducted with the plain PES membrane as well as modified membranes at a transmembrane pressure (TMP) of 1 bar. As seen in Figure 13, all of the modified membranes showed a permeate flux that is higher than the plain membrane. For instance, the plain PES membrane showed an average permeate flux of 323.2 ± 13.2 LMH while PES membranes modified with various loadings of CAR in the dope solutions (M2–M8) showed an average permeate flux ranging between 550 ± 10.2 and 1429 ± 9.0 LMH at a TMP of 1 bar. The addition of CAR to casting solutions was found to enhance the permeate flux at all loadings. In fact, the higher the loading of CAR is, the greater the permeate flux of PES/CAR membranes. The highest permeate flux was encountered at a CAR loading of 4 wt.%. The CAR incorporation onto the casting solutions was observed to increase the permeate flux from 323.2 ± 13.2 to 1429 ± 9.0 LMH, which accounts for a more than three-fold enhancement. The improvement in water flux could be attributed to the enhanced membrane porosity and hydrophilicity. The water flux results are in good agreement with the SEM characterization data, which shows that CAR addition to the dope solutions led to the formation of more porous membranes (Figure 7). Moreover, the characterization of the contact angle of the plain PES as well as PES/CAR membranes showed that the modified membranes were more hydrophilic than not only plain PES membranes but also commercial membranes such as PES 10 kDa from Snider and PES 50 kDa and PES 150 kDa membranes from Mann+Hummel.

The water fluxes of the prepared membranes in the present work were found to outperform the PES membranes modified with other additives reported in the literature. For instance, Zhang et al. [60] prepared modified PES membranes by adding vinyl triethylene (b-methoxy ethoxy) silane (VTMES) to the casting solutions. It was observed that the optimum membrane performance during filtration of humic acid solutions (5 ppm) was obtained at 1 wt.% loading of VTMES into the casting solutions with a rejection efficiency of 97% and permeate flux of 326 LMH. PES UF membranes, modified with (gamma-mercaptopropyl)trimethoxysilane prepared by Rezvanie et al. [61], showed a pure water flux below 100 LMH with the flux dropping to less than 70 LMH after filtration for 60 min. Moreover, PES membranes modified with graphene oxide by Marjani et al. [62] showed a maximum water flux below 60 LMH, corresponding to a PES membrane with 4 wt.% graphene oxide loading.

#### 3.8.2. BSA Filtration

**Figure 14:**
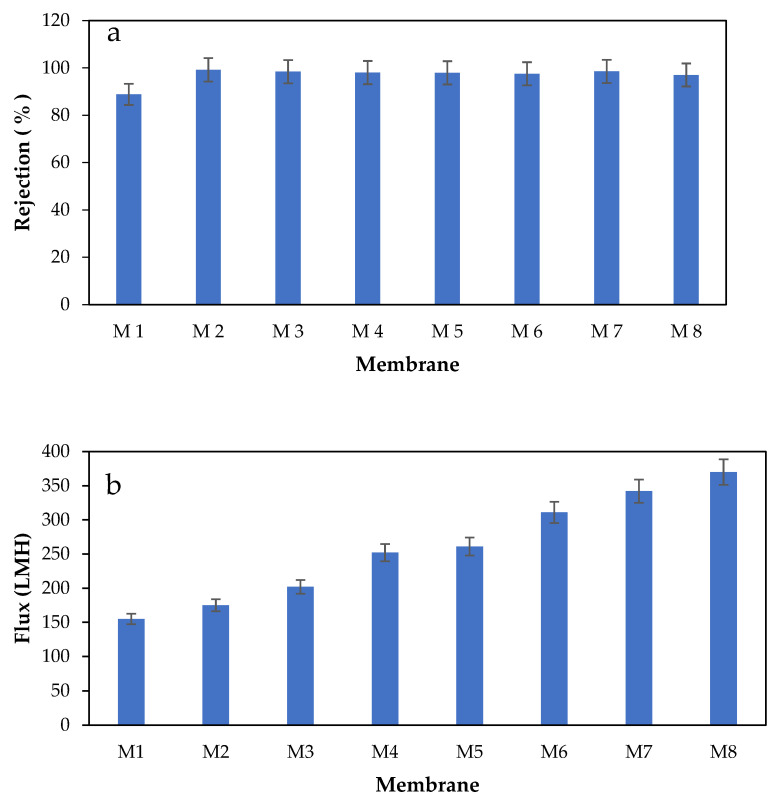
Rejection (**a**) and permeate flux (**b**) of PES membranes incorporated with various loadings of CAR in casting solutions during filtrations with 300 mg/L BSA (operating pressure: 1 bar). (M1: pure PES, M2: 0.1 wt.% CAR, M3: 0.3 wt.% CAR, M4: 0.5 wt.% CAR, M5: 0.75 wt.% CAR, M6: 1 wt.% CAR, M7: 2 wt.% CAR, and M8: 4 wt.% CAR).

The performance of the prepared membranes was tested by conducting filtration experiments using BSA solutions (300 mg/L). Figure 14a illustrates the rejection efficiencies while Figure 14b shows the permeate flux of the plain as well as PES/CAR membranes during filtration of BSA solutions. The plain PES membrane showed a rejection efficiency of 88.5 ± 4.2 (%) while the modified membranes showed a removal efficiency above 97%. As seen, all modified membranes demonstrated consistently higher BSA rejection rates when compared with the plain PES membrane, obviously due to the formation of a hydration layer on the surface and in the pores of more hydrophilic PES/CAR membranes, which enhanced the membrane rejection [63]. The higher negative zeta potential of the PES/CAR membranes might also contribute to stronger electrostatic repulsion between the membrane surface and the negatively charged BSA macromolecules at feed. It is worth pointing out that during filtration experiments, the pH of the feed BSA solutions was pH 6.4, which is higher than the point of zero electric charge of pH at pH 4.7 [64].

Moreover, the permeate flux of the modified membranes ranged between 175 ± 5.1 and 370 ± 18.4 LMH while that of the plain PES membrane was 155 ± 4.1 LMH. The addition of 0.1 and 4 wt.% CAR to PES membranes was observed to increase the permeate flux during filtration of BSA by 13% and 138%, respectively. The enhancement in the flux of the PES/CAR membranes could be attributed to their higher porosity, pore size, and hydrophilicity.

The performance of the modified membranes was found to outperform some of the membranes reported in the literature. For instance, Liu et al. [65] modified PES UF membranes with an amphiphilic copolymer from poly(N-methyl-D-glucamine) for the development of antifouling membranes. The modified membranes were tested with BSA aqueous solutions and showed a permeate flux below 25 LMH and rejection efficiencies below 70%.

#### 3.8.3. Membrane Antifouling Properties

The FRR of the prepared membranes was assessed by filtrating the DI water first, followed by aqueous BSA solutions (300 mg/L), and finally with DI water again. The FRR values of all the membranes were calculated using Equation (5). The FRR is usually used to indicate the antifouling capability of the membranes. A high FRR value indicates that a membrane was able to recover a significant portion of the initial water flux following BSA filtration. The analysis on the FFR values showed that the FRR values of the modified PES membranes were 20–40% higher compared with the pure PES membranes. The highest FRR corresponded to the M6 membrane with a CAR loading of 1 wt.%. This can be attributed to the enhanced hydrophilicity, and negative surface charge of the modified membrane, which reduced the interaction between the membrane surface and BSA molecules in water. High fouling resistance is crucial to maintaining the membrane’s long-term effectiveness and lowering the need for frequent cleaning and maintenance.

**Figure 15:**
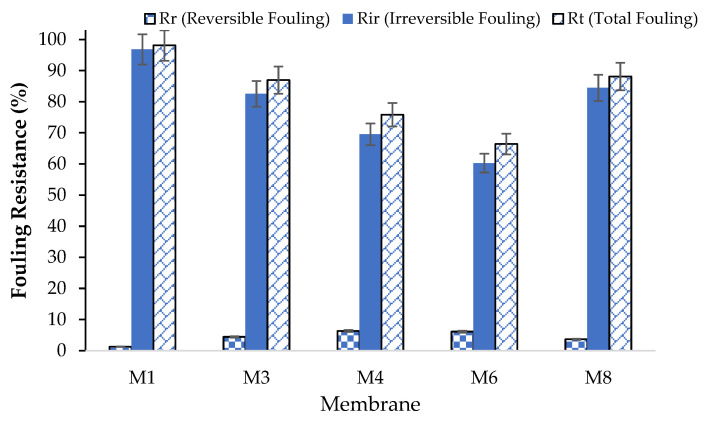
Rt, Rr, and Rir of plain membrane and membranes incorporated with various loadings of CAR in dope solutions during filtration of BSA solutions at 300 mg/L; TMP: 1 bar, pH: 6.8. (M1: pure PES, M3: 0.3 wt.% CAR, M4: 0.5 wt.% CAR, M6: 1 wt.% CAR, and M8: 4 wt.% CAR).

Reversible membrane fouling occurs when the membrane’s foulants have a low affinity to attach themselves to the membrane surface. Hydraulic cleaning procedures can effectively remove this type of fouling. On the other hand, irreversible fouling takes place when foulants attach themselves forcefully to the membrane’s surface. The removal of this kind of fouling using conventional cleaning techniques is a bit challenging. Figure 15 shows the total, reversible, and irreversible fouling which were evaluated using Equations (6)–(8). As seen, the PES/CAR membranes demonstrated a reduction in the irreversible and total fouling. For instance, the incorporation of CAR in the M6 sample was observed to reduce the irreversible and total fouling of the plain PES membrane from 96.8% and 98% to 60% and 66.4%, respectively. These values correspond to a reduction in the irreversible and total membrane fouling by 38% and 32%, respectively. The enhancement in the antifouling properties of the prepared membranes toward BSA can be attributed to the increase in the hydrophilicity and surface charge of the PES/CAR membranes compared with the plain PES membrane. This enhanced antifouling resistance of the prepared membranes would be highly beneficial for the separation of protein-containing streams in biotechnology and in the treatment of effluent in the dairy industry.

#### 3.8.4. Membrane Compaction

**Figure 16:**
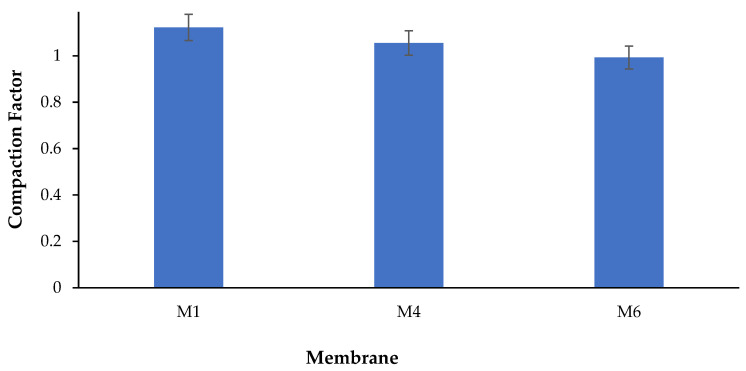
Compaction factor for M1, M4, and M6 membrane samples at an operating pressure of 1 bar. (M1: pure PES, M4: 0.5 wt.% CAR, and M6: 1 wt.% CAR).

Membrane compaction can be observed after the compression of porous membranes following their exposure to pressure which reduces the membrane thickness and porosity. The overall effect of membrane compaction can be manifested by the flux drop which is proportional to the pressure across the membrane. In the present work, the determination of the compaction factor (CF), which is often used to estimate how the porous structure of a membrane changes when pressure is applied, was performed. Equation (4) was used to estimate the CF while Figure 16 shows CF values for the pure and modified membranes. The higher the CF is, the higher the flux drop due to membrane compaction. As shown in Figure 16, the plain PES membrane (M1) had a CF value of 1.1 ± 0.05, while the CAR-containing membranes (M4 and M6) had a CF of 1.05 ± 0.04 and 0.99 ± 0.04, respectively. The modified membranes showed a lower CF which indicates higher resistance to membrane compaction.

Membranes with higher CAR loadings demonstrated higher compaction resistance. During membrane compaction, the thickness of the top layer of the membrane frequently gets reduced as it merges with the underlying sublayer. Furthermore, the membrane pore walls can undergo compaction and approach each other, resulting in a reduction in pore size and a decrease in flux. Adding CAR to the PES matrix improves the membrane resistance to compaction, as shown by lower CF values for the PES/CAR membranes. These findings correlate well with DMA results, which showed enhanced mechanical properties of the PES/CAR membranes (Figure 12). The enhancement in the mechanical properties and the resistance to membrane compaction following the addition of CAR macromolecules to PES membranes was found to agree with work reported in the literature. For instance, the incorporation of AG into PES casting solutions produced UF membranes with a 31% increase in tensile stress compared with bare PES membranes [34]. This can be attributed to the arrangement of amphiphilic AG macromolecules to anchor themselves to the PES matrix. The linking between the hydrophobic end of AG macromolecules with the hydrophobic PES matrix was reported to result in strengthening the porous matrix of PES membranes [34].

## 4. Conclusions

The use of CAR as an additive to the casting solutions for the preparation of novel PES membranes by the NIPS method was presented for the first time in this work. The investigation of the effect of CAR on the properties and performance of novel PES membranes was reported at various loadings of CAR (0.1–4.0 wt.%) into casting solutions in DMSO. DMSO is a bio-based solvent that has less toxicity compared with DMA, NPM, and DMF, which are typically used for the preparation of UF PES membranes. PES/CAR membranes were observed to demonstrate higher porosity, hydrophilicity, surface charge, and pore size when compared with the bare PES membrane due to the characteristic hydrophilic nature and inherited pore-forming properties of CAR. The filtration experiments with BSA solutions showed that PES/CAR membranes had higher antifouling properties compared to plain PES membranes. The addition of CAR to PES casting solutions resulted in enhancing the FRR of PES/CAR membranes by 20–40% compared with the plain PES membrane. Moreover, the irreversible and total fouling for the PES/1 wt.% CAR membrane was reduced by 38% and 32%, respectively, compared to the plain PES membrane. Notably, the modified membranes increased the Young’s modulus by about 1.5-fold when compared with the bare PES membrane. The findings of the present study indicate that CAR can be used as a novel pore-former, hydrophilizing, and antifouling agent, as well as an enhancer of the mechanical and rejection properties of the PES membranes.

## Figures and Tables

**Table 1:** Composition of the dope solutions for the membrane casting.

Membrane	CAR (%)	PES (%)	DMSO (%)
M1	0	16	84
M2	0.1		
M3	0.3		
M4	0.5		
M5	0.75		
M6	1		
M7	2		
M8	4		

**Table 2:** Average surface roughness of PES membranes doped with various loadings of CAR onto casting solutions. (M1: pure PES, M3: 0.3 wt.%, M4: 0.5 wt.%, M6: 1 wt.%, M7: 2 wt.%, and M8: 4 wt.% CAR.)

Membrane	Ra (nm)
M1	2.46 ± 3.11
M3	2.27 ± 2.84
M4	2.14 ± 2.80
M6	1.44 ± 1.82
M7	2.02 ± 2.60
M8	2.98 ± 3.74

## Data Availability

The original contributions presented in this study are included in the article. Further inquiries can be directed to the corresponding author.
